# Sectional matrix solutions: the distorted truth

**DOI:** 10.1038/s41415-021-3608-5

**Published:** 2021-11-12

**Authors:** Oliver Bailey

**Affiliations:** Newcastle University School of Dental Sciences, Newcastle upon Tyne, NE2 4BW, UK

## Abstract

Sectional matrix techniques offer more predictable solutions to achieving contact areas when placing direct interproximal posterior composites than circumferential matrix techniques, resulting in reduced reported complaints of food packing from patients. Despite this, a large majority of UK dentists and therapists don't currently use them. Sectional matrix systems are technique-sensitive to use, which can be a barrier to implementation for inexperienced users. The matrices can easily distort during their placement and stabilisation and when placing the restorative material. This can result in unwanted, clinically relevant problems in the resulting restorations, some of which may not be discernible once they have occurred. This paper explores the advantages and disadvantages of sectional matrices and the processes and techniques involved in their use, before discussing the potential for distortion at each step. It offers solutions to some of the commonly seen problems which will provide more predictable outcomes for those already using these techniques and encourage non-users to add them to their armamentarium.

## Key points

- Considers the advantages and disadvantages of using sectional matrix techniques.
- Explores the key elements in selecting, placing and stabilising sectional matrices, whilst separating teeth sufficiently to achieve a contact with the adjacent tooth.
- Describes the mechanisms of sectional matrix distortion and suggests techniques to minimise these, resulting in predictable patient-centred outcomes.

## Introduction

Posterior composite restorations generally perform less well than amalgam restorations,^1^^,^^2^ especially in primary care.^3^ Clinicians are much less confident in placing posterior composite restorations, especially in difficult situations due to their increased technique sensitivity.^4^ Techniques classically taught at undergraduate level to rebuild the lost interproximal portion of a tooth involve the use of a circumferential matrix band with a matrix holder (for example, Toffelmire and Siqveland) and a wooden wedge.^5^ This is by far the most commonly used technique in UK primary care for the placement of both amalgam and composite restorations.^6^ Amalgam is actively placed, in that it must be firmly packed and compacted into the cavity to form the restoration. This packing, alongside the firm placement of a wooden wedge,^7^ puts pressure on a pre-burnished matrix, which favours the formation of a contact point (or more accurately, contact area). Creating a contact area between the restored tooth and adjacent tooth is important to prevent food impaction in the area, often being uncomfortable for patients and a common cause for complaint.^6^^,^^8^ It can also potentially increase the risk of further caries and periodontal disease, though evidence commonly cited to support this contention is cross-sectional and therefore not robust.^9^^,^^10^

Composite on the other hand is passively placed, in that there is limited force imparted and maintained during placement before curing (most commonly) with a light. It also shrinks when undergoing polymerisation.^11^ This explains the tendency of composites to perform less well than amalgam in terms of contact point creation and prevention of food impaction,^6^^,^^7^ even with so-called 'packable composites'.^11^ The consistency of the composite material can have an effect on contact point formation, however, with paste-like formulations performing better than flowable formulations.^12^ When restoring proximal cavities where only one surface is lost, the circumferential matrix band also has to pass through the intact contact point at the other side of the tooth, which will result in tooth displacement, further reducing the chances of achieving a contact area between the resulting restoration and adjacent tooth.^13^

**Fig. 1:**
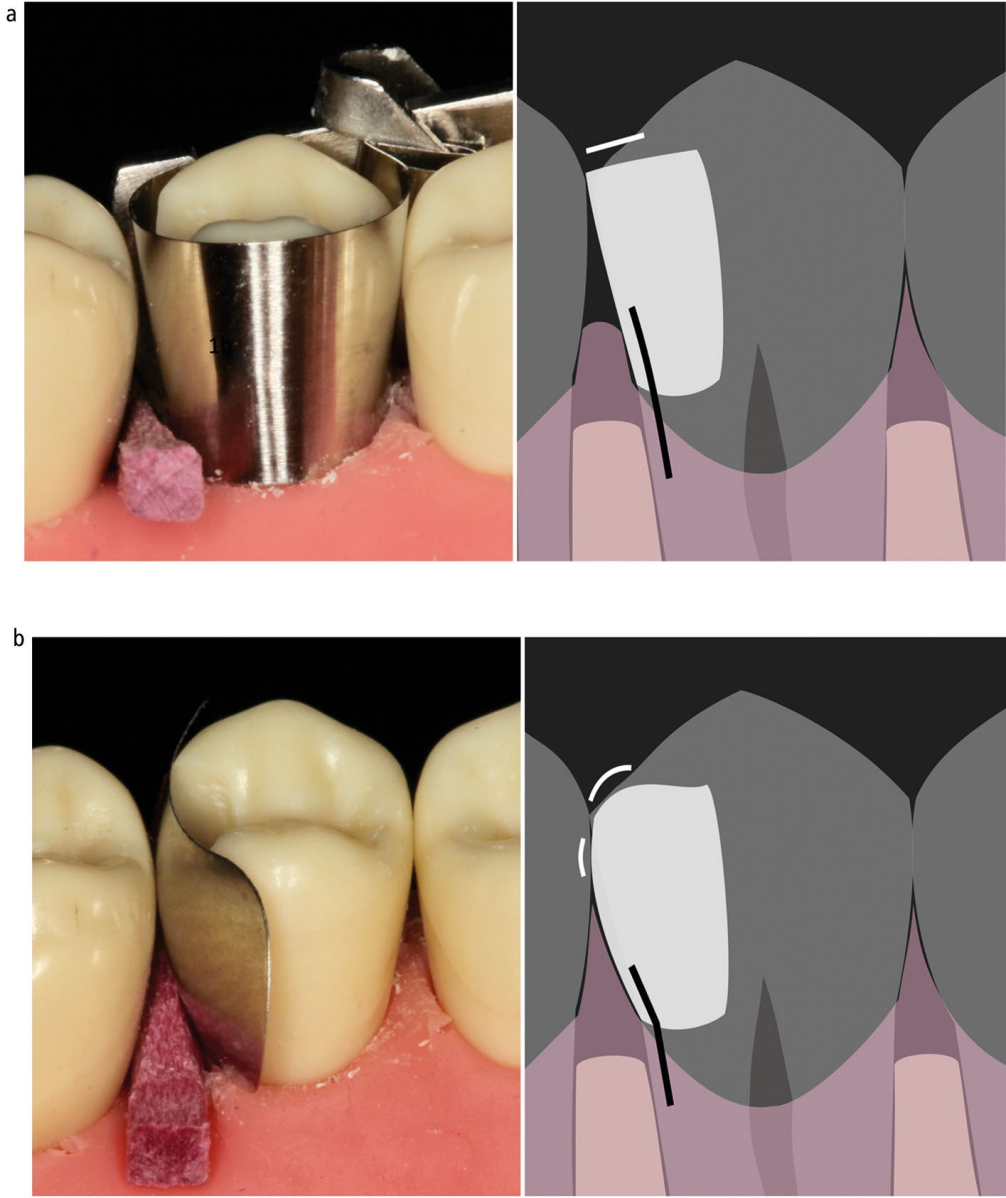
a) Contact 'point' placed above point of maximum convexity of adjacent tooth if achieved (often not). Marginal ridge laterally positioned (to maintain contact) and therefore thin, unsupported and susceptible to fracture. Embrasure flat resulting in a tendency for floss to catch and shred. Non-anatomical 'flat' cervical emergence coupled with high contact point. Tendency to interproximal dead space allowing food impaction. (Wedge position limited by matrix holder). b) Contact area broader. Marginal ridge anatomically positioned and well supported. Embrasure convex resulting in supported anatomical marginal ridge and allowing easy, unimpeded floss access. Anatomical cervical emergence. Papilla fills interproximal area

Sectional, pre-contoured (more anatomically shaped) matrices were developed to overcome these problems. They are classically used in combination with a separating ring, which provides separation of the teeth and stabilises the matrix coronally, favouring the formation of a contact area.^13^^,^^14^^,^^15^ Circumferential matrices are tightened around the tooth and are therefore described as being placed actively. This active placement does potentially confer an advantage over sectional matrices, which are passively placed (not tightened), in that it stabilises the matrix, both cervically and coronally, resulting in reduced formation of overhangs, especially bucco-palatally.^16^ This is also very useful for teeth which are heavily broken down.^11^ Circumferential matrices have many relative disadvantages however, in that it is very difficult to recreate an anatomical emergence^12^^,^^15^ which makes achieving a contact area difficult.^14^^,^^17^ They also result in an inferior morphological contact with reduced contact tightness.^15^^,^^17^ Even if a contact is achieved, it is more akin to a single point of contact rather than a broad area and is positioned in a non-anatomical, more coronal position.^18^ This then often results in a laterally positioned, unsupported marginal ridge form which is more susceptible to fracture^19^ and a flatter emergence form from the embrasure with the potential to catch and shred floss, resulting in a patient complaint. The combination of a higher or non-existent contact point and a flatter, non-anatomical cervical emergence lead to an increased chance of incomplete papilla infill.^20^ This leads to dead space (seen as black triangles) below the contact point which can predispose to food impaction, as evidenced by the increased reported food packing when using circumferential compared with sectional matrices for posterior composites in primary care (Fig. 1).^6^ The matrix holder also often limits access for wedge placement, which can have an impact on their efficacy (see later).

**Fig. 2:**
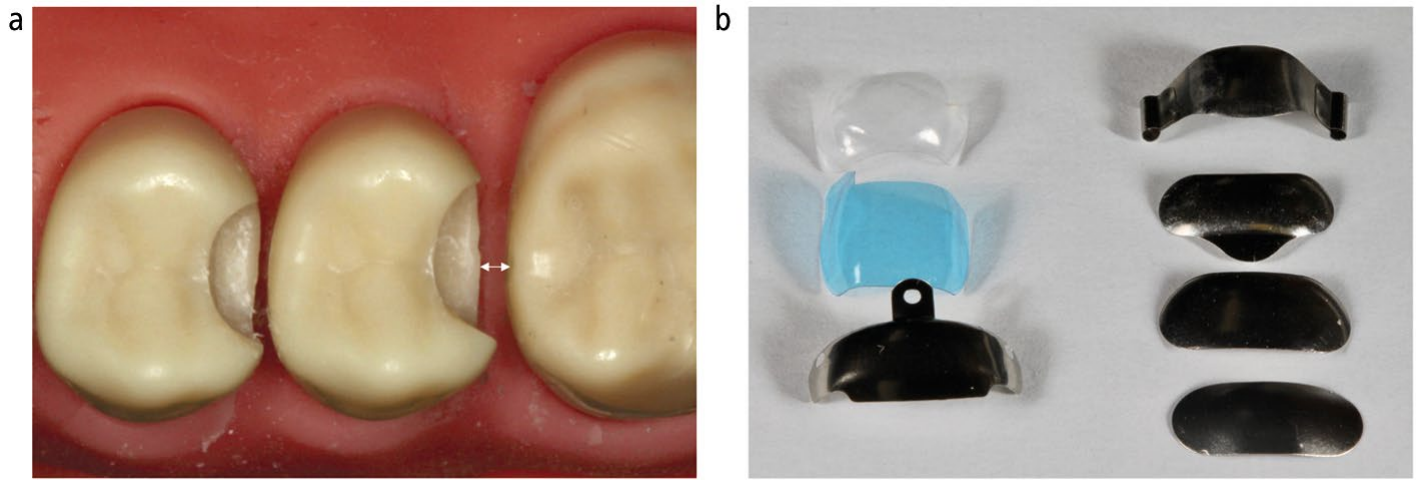
a) Distance from cavity to adjacent tooth important in matrix selection. b) Flexible matrices on left, more rigid matrices on right, available in a variety of shapes and sizes allowing appropriate selection in each individual situation (see Figure 3)

Recent research suggests that the use of sectional matrices for placing posterior composites where an interproximal surface has been lost is low in the UK,^6^ despite the advantages previously described and their use being referred to as a gold standard of care.^5^ There is a fairly steep learning curve involved in using sectional matrices however, and they are quite technique-sensitive to place, such that inexperienced operators preferred to use circumferential matrices even when obtaining better clinical outcomes.^14^ Sectional matrices are available in different material constructions, opacities, heights, widths, rigidities and emergence profiles (Fig. 2), with a bewildering array of associated equipment, which can make selection difficult for any dentist with limited experience in this area.

**Fig. 3:**
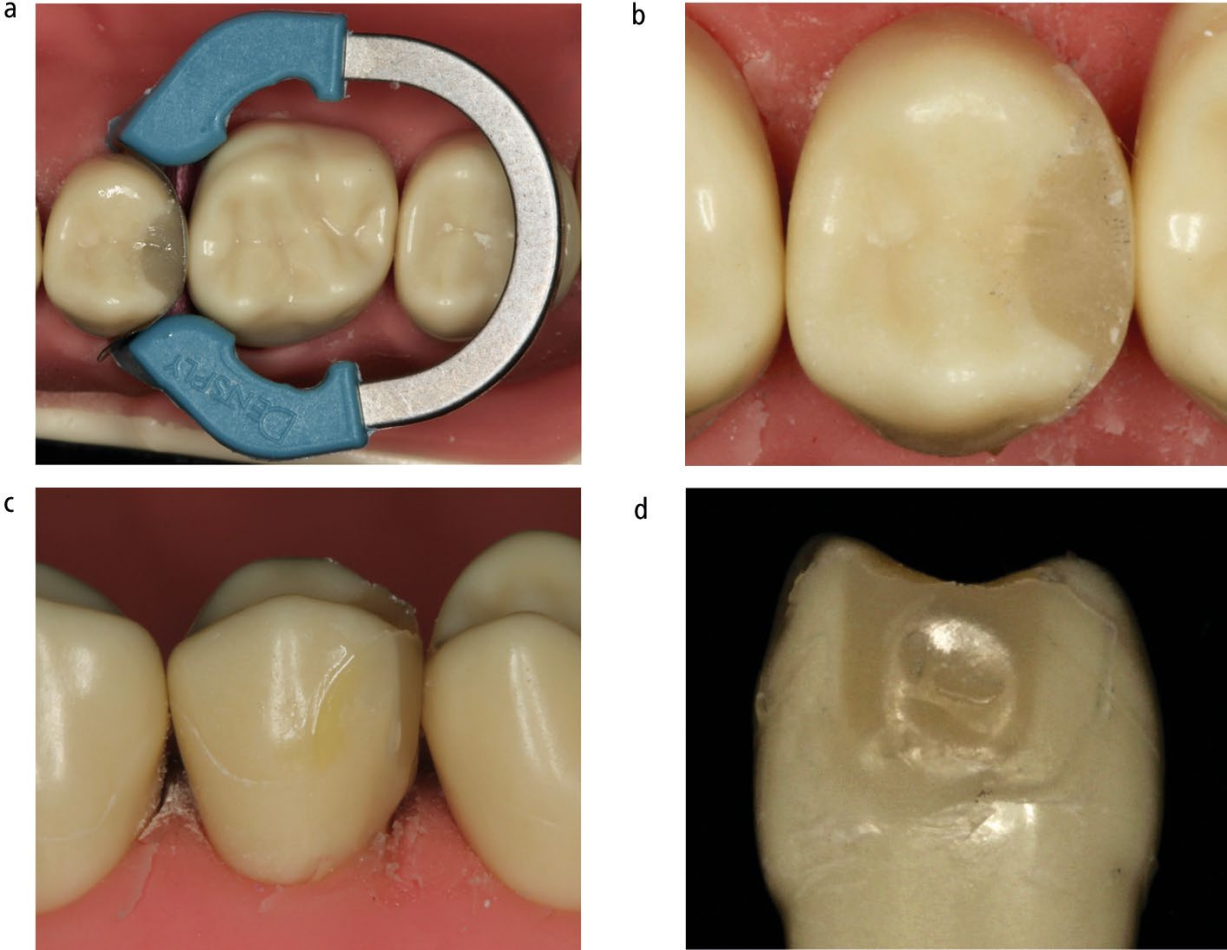
a, b, c, d) Tooth restored using sectional matrix and separating ring. Very tight contact, with peripheral ledging and concavity at contact area and beyond. Concavity only evident when tooth removed

While sectional matrix techniques using separating rings can result in the predictable establishment of contact areas,^13^^,^^14^^,^^15^ they have been shown to result in surface concavity in the restoration at the contact area, which is much less of a problem with circumferential matrices.^21^ A concavity in the restored surface at the contact area will be inaccessible to cleaning and tend to harbour biofilm and is often not identifiable clinically^21^ (Fig. 3). Given that composite materials favour growth of a cariogenic biofilm on their surface,^22^ this could potentially result in the initiation and progression of caries in the proximal surface of an unrestored adjacent tooth.

This paper will explore why this, along with the increased propensity for overhangs occurs and potential solutions to these problems.

## Sectional matrix technique

### Aim

The aim of a sectional matrix is to produce a cleansable, anatomical restoration with a smooth convex surface, which is continuous with the remaining tooth structure and has a contact area at the level of the maximum convexity of the intact adjacent tooth.

This is achieved by fulfilling the objectives related to sectional matrices summarised in Table 1. These will be explained in turn, before discussing matrix distortion, its possible sequelae and how available materials and techniques can influence this.

**Table 1:** A summary of objectives, methods used to achieve them and how they can be affected, when selecting and placing a sectional matrix

Objective	Methods used/how objective can be affected
A well-adapted matrix in contact with the adjacent tooth at the point of maximum convexity	Affected by cavity design, dimensions and configuration, matrix selection and placement, stabilisation techniques, restorative material placement
Cervical seal and stability	Methods: wooden and plastic wedges, mechanical separators (eg Elliott), adjunctive use of PTFE teflon tape, 'Teflon floss' technique
Separation of the teeth greater than or equal to the width of the matrix used	Methods: wedges, separating ring, Elliott separators
Coronal stability	Methods: separating ring (active), unbonded flowable resin (passive). (Also affected by matrix material, rigidity and shape)
An undistorted matrix	Potentially affected by all of the above

### Well-adapted matrix in contact with the adjacent tooth

**Fig. 4:**
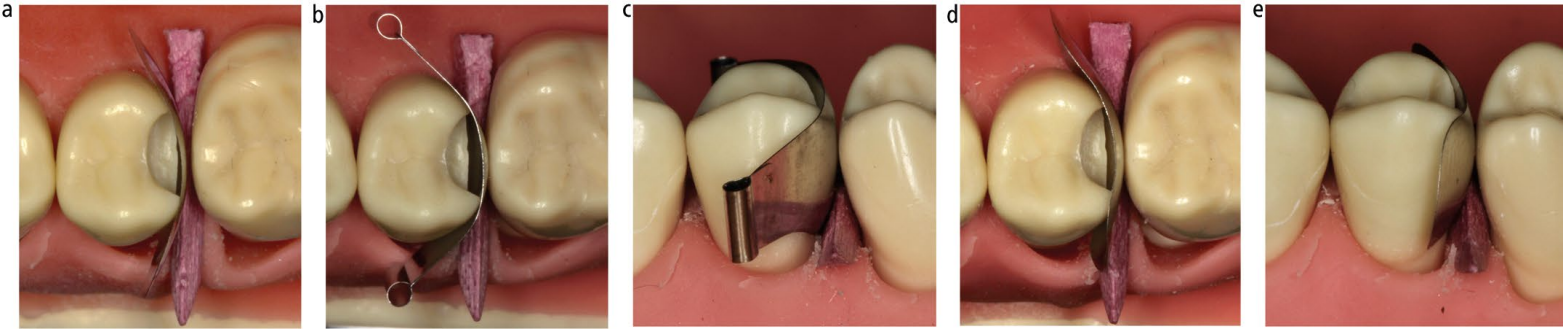
a) Matrix with insufficient occluso-cervical curvature not contacting adjacent tooth following appropriate wedging. b, c) Matrix with increased occluso-cervical curvature resulting in acceptable positioning of contact (potentially slightly coronal). However, adaptation occlusal to the contact area is sub-optimal, potentially requiring increased finishing. Repositioning the matrix more apically may address these issues. This may require adjustment of the matrix cervically. d, e) Matrix with increased occluso-cervical curvature resulting in good positioning of contact and improved adaptation occlusal to the contact. This is the most appropriate matrix selection from these three matrices in this specific situation. Note however that the increased curvature may lead to increased potential for placement distortion

There are a wide variety of sectional matrices available (Fig. 2) and selection of the most appropriate one can influence the resulting marginal overhang, with flexible types performing better than malleable, soft types.^16^ Matrix choice will primarily be governed by the shape of the tooth, the shape and depth of the cavity and its proximity to the adjacent tooth (Fig. 2). A matrix should be selected such that it extends beyond the extent of the cavity and can be engaged and stabilised. The maximum convexity of the matrix should be positioned against and in contact with the maximum convexity of the intact adjacent tooth to create an appropriate contact area (Fig. 4). The matrix should be able to be placed passively, unimpeded by contact with the adjacent tooth.

The mesio-distal matrix curvature and curvature occlusal to the contact area will also affect the marginal adaptation and therefore potential ledge formation in the resulting restoration, which will impact on the need for finishing of the restoration (Fig. 4). Matrices may need to be modified; for example, by trimming them cervically, to optimise adaptation.

### Matrix stabilisation and seal - cervical

The matrix has to be stabilised and sealed at the base of the cavity. These elements optimise the adhesive bonding process and prevent ledge formation in the resultant restoration. Once formed, ledges in this area can be difficult to remove. If left, ledges can be difficult to clean, resulting in biofilm accumulation, potentially resulting in secondary caries and periodontal disease.^23^ Composite resin and resin adhesives have been shown to support and favour the development of a cariogenic biofilm on their surfaces,^22^^,^^24^ which could exacerbate the potential for secondary caries in ledged composite restorations.

**Fig. 5:**
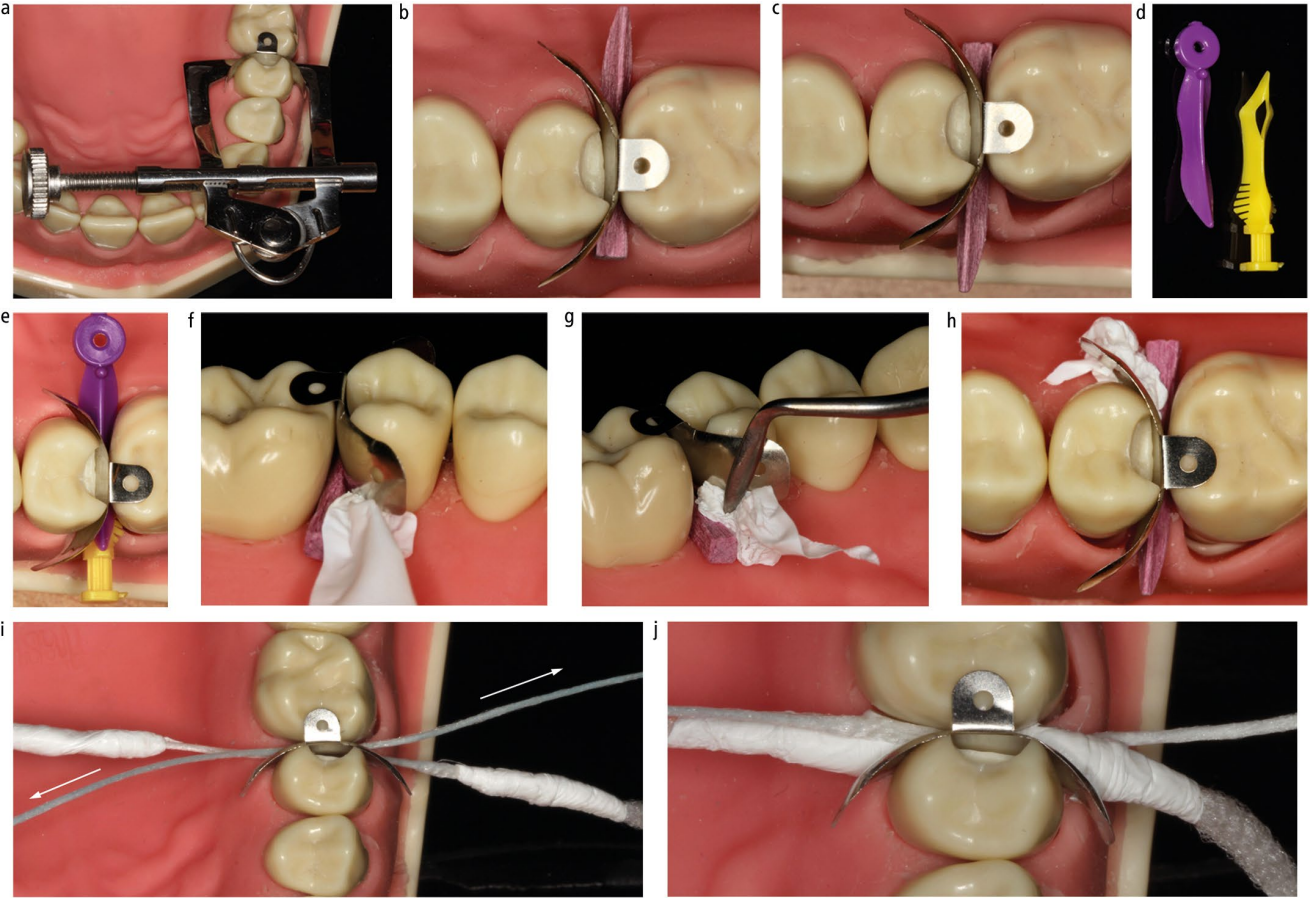
a) Elliott Separator. b, c) Wedge placement direction affecting cervical seal. d, e) Plastic contoured wedges allowing engagement around cervical curvature and synchronous placement from both sides providing improved cervical adaptation. f, g, h) Packing of PTFE tape to provide cervical seal and stabilization of matrix. i, j) Teflon-floss technique. Teflon-floss pulled simultaneously in directions of arrows creating seal

Wedges are most commonly used for cervical stabilisation of a matrix, though mechanical separators (for example, Elliott) or the 'Teflon-floss' technique may be used as alternatives (Fig. 5).^17^^,^^25^ Insertion of the wedge from the buccal or palatal can have varying effects on the cervical stability and seal achieved (Fig. 5). Plastic wedges are available in multiple designs, though the majority are contoured and flexible with the aim of engaging around the interproximal curvature (Fig. 5) in an attempt to seal the whole base of the cavity. They also generally have concavities on their undersides, which allow them to sit over the papilla with a low profile^26^ and facilitates their insertion from each side of a cavity to further obtain a better cervical seal (Fig. 5). Polytetrafluoroethylene (PTFE) tape can also be applied in conjunction with a wedge to stabilise and seal any open area at the base of a cavity (Fig. 5). The Teflon-floss technique involves winding PTFE tape around two pieces of knotted floss and simultaneously drawing them in from both sides of the matrix, adapting the matrix to the base of the cavity^25^ (Fig. 5). This is purported to result in a reduced tendency to break the dental dam seal than when using wedges, which can pick up and drag the dam, opening up gaps. The Elliott Separator is suggested to have a similar advantage, but can be difficult to position and stabilise. Likewise, there is reduced control over the positioning of the Teflon-floss due to its lack of rigidity, which could potentially move the matrix and it may therefore be better used after a separating ring has been placed,^25^ which can result in its own issues (discussed later).

Following this process, the matrix should be in contact with the adjacent tooth. If it isn't, a different matrix should be selected with more cervico-occlusal curvature (Fig. 4).

### Tooth separation

When placing an interproximal composite with the aim of creating an interproximal contact between the restored and adjacent teeth, the thickness of the matrix is critical to consider. The matrix is removed after placement of the restoration and would therefore result in a gap between the restored tooth and the adjacent tooth, if these teeth aren't separated before placement of the restoration. The teeth can be transitorily moved apart by virtue of the compressibility of their periodontal ligaments before placement, thus allowing the formation of a contact when the matrix is removed.^7^ This can be achieved by using wooden wedges, separating rings, or Elliott Separators,^7^^,^^13^^,^^17^ though whether this is the case for the different designs of plastic wedges or the Teflon-floss technique is currently uncertain. Wooden wedges can predictably provide lasting separation of 50 microns,^7^ which is sufficient to accommodate most metal matrices available, but some clear matrices are 75 microns thick, therefore separation with a wooden wedge alone would not be recommended.

### Matrix stabilisation - coronal

The matrix has to be stabilised coronally. Lack of coronal stabilisation can lead to distortion of the matrix during composite placement.^21^ Coronal stabilisation is also important to minimise ledge formation, though this is also affected by the adaptation of the matrix. Coronally located overhangs are much more accessible for finishing than those at the base of the cavity when the cavity is appropriately designed (see later), so they aren't as critical to avoid. Coronal stabilisation can be active, where a force is applied to the matrix using a separating ring, or passive, where the stabilisation is provided without an applied force, through the use of unbonded flowable composite. More rigid matrices have a tendency to self-stabilise to a degree, whereas more flexible ones do not.

### Matrix distortions

When a force is applied to a sectional matrix, distortions can occur. They can arise during placement, separation and (cervical and coronal) stabilisation of the matrix and when placing the restorative material.

Sectional matrix distortions can occur peripherally and/or centrally, with different potential sequelae. Peripheral gaps or distortions commonly result in ledged restorations, or failure to seal the base of the cavity, whereas central distortions often lead to concavities at the contact area (Fig. 3). Distortions can also result in the loss of a contact.

### Placement distortion

**Fig. 6:**
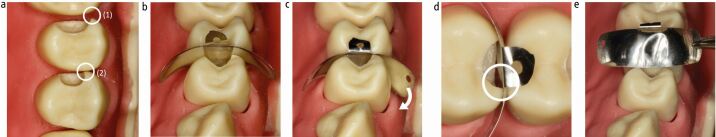
a, b, c, d, e) Resultant placement distortion of matrix shown

**Fig. 7:**
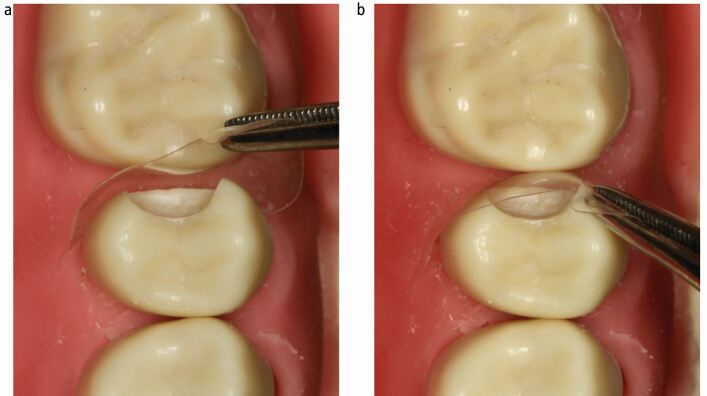
a) Clear matrix distorted on insertion. b) No permanent deformation when seated

A cavity design where the proximal contacts are cleared both cervically and bucco-palatally is critical to facilitate passive matrix placement (Fig. 3). This helps to avoid distortion of the matrix during placement (Fig. 6). It also has the added benefits of placing the tooth-restoration interface away from a contact area, allowing access to the margin for optimal finishing of the restoration and subsequent cleaning of the restored tooth, thereby potentially reducing the risk of future caries development. Distortion can also occur around the critical contact area during placement of the matrix, as this is the most bulbous part of the pre-contoured matrix and therefore the part most likely to be distorted by contact with the adjacent tooth during placement (Fig. 7). Distortion of metal matrices during placement is more likely to be permanent than with clear matrices. More curved matrices are also more susceptible to this distortion. This results in an altered matrix shape. Clear matrix distortions are more able to be resolved once positioned, due to their increased elasticity (Fig. 7).

### Stabilisation distortion

As the sectional matrix is passively placed and often not stabilised before placing the wedge, it can have a tendency to move. This potentially results in distortion of the matrix peripherally and/or centrally, or moving the matrix to an incorrect position. Stabilising the matrix from the occlusal with a finger or thumb while placing the wedge can generally overcome this tendency.

**Fig. 8:**
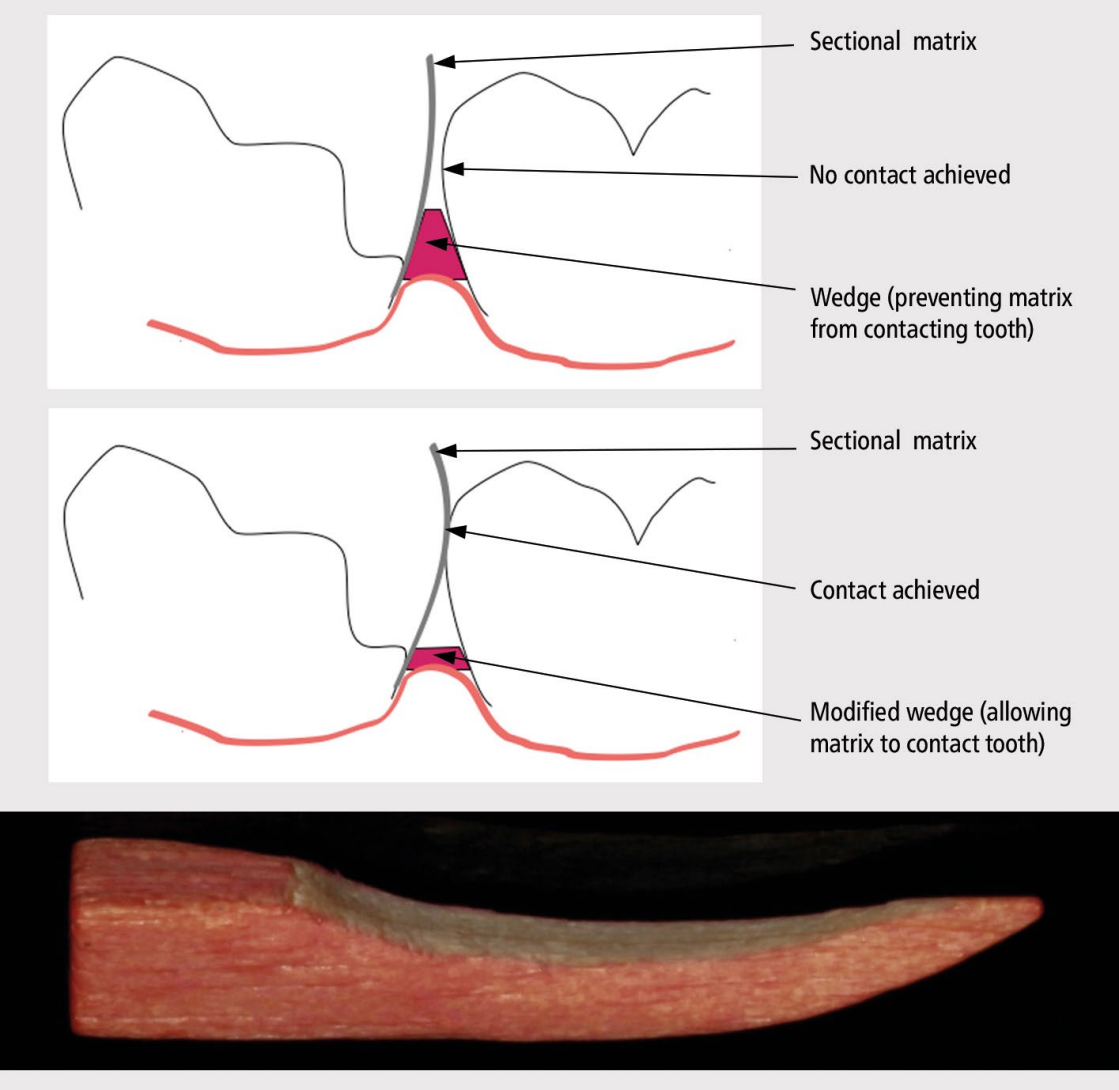
Importance of wedge modification in facilitating contact area establishment

It is important to ensure that the wedge is inserted below and subsequently lies below the base of the cavity.^26^ Fulfilling these objectives help to obtain a seal and prevent both peripheral and central matrix distortion. Appropriate management of the papillae to achieve this is important where the cavity margin lies sub-gingivally.^26^ Wooden wedges may require modification to prevent their protrusion coronally above the base of the cavity (Fig. 8). This process can be performed with a bur and has previously been pictorially demonstrated.^26^ When inadequately performed, the wedge can impinge on the matrix, which in turn can prevent the recreation of an anatomical emergence and subsequent formation of a contact area in the resulting restoration (Fig. 8). The Teflon-floss technique is also prone to this distortion because of its own propensity to distort, which offers an advantage in adapting the matrix to the base of the cavity, but a lack of control over positioning (Fig. 5e). Ideally, the wedge would engage the tooth at the level of the cavity margin, preventing the potential for gaps to open up when subsequently applying forces to the matrix, but this is unrealistic and other solutions should be sought to minimise peripheral stabilisation distortion (see later). Wedges are therefore ideally tried in to check their adherence to the achievable goals before insertion of the matrix.

**Fig. 9:**
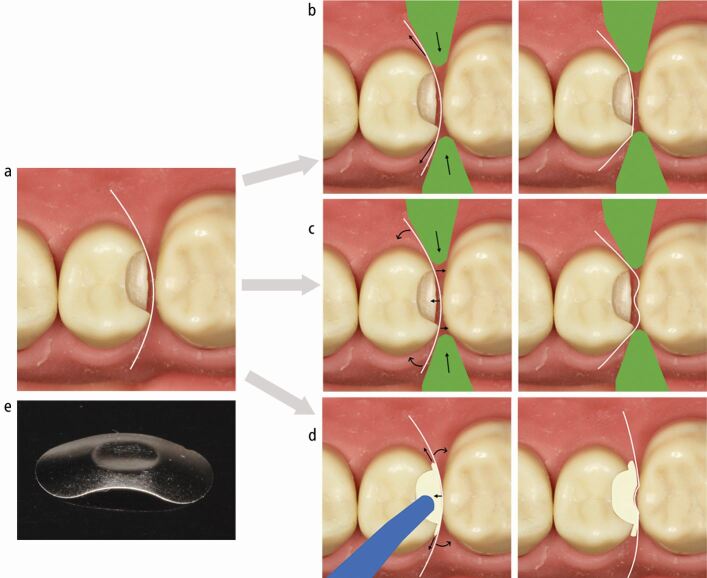
Mechanisms of distortion. a) Undistorted matrix. b) Coronal stabilisation distortion. Separating ring. Loss of contact. c) Coronal stabilisation distortion. Separating ring. Tenting. Peripheral and central distortion. d) Extrusion distortion. Composite dispensed. Peripheral and central distortion. e) Central matrix distortion

**Fig. 10:**
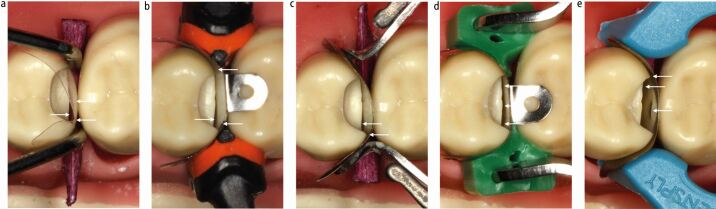
a, b, c, d, e) Different matrix/wedge/separating ring combinations resulting in various distortions (arrows) at the base of the box, bucco-palatally and in the contact area

**Fig. 11:**
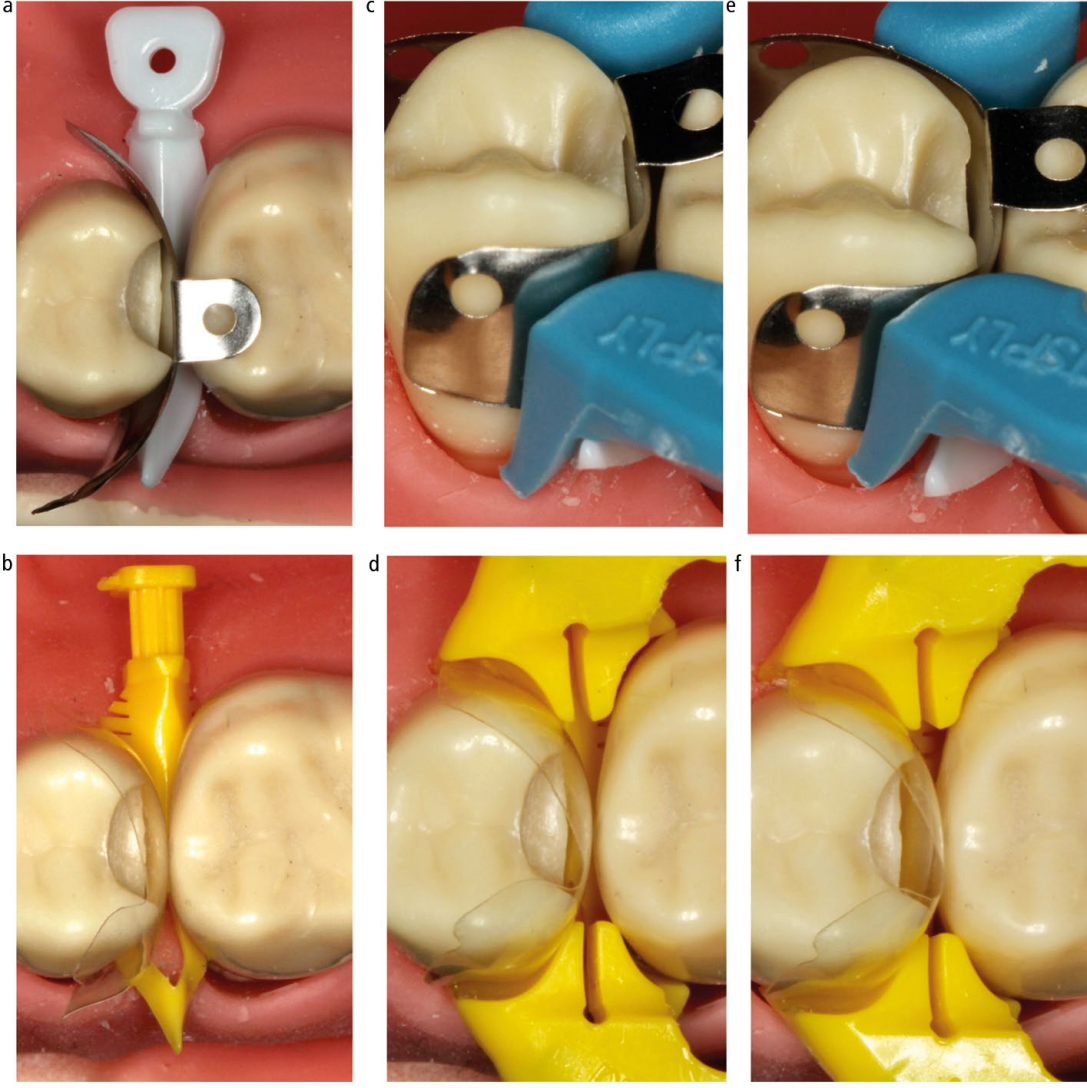
Stabilisation distortion. a, b) Undistorted, well-positioned matrices. c, d) Separating ring resulting in potential loss of contact. e, f) Same separating ring/matrix/wedge combination resulting in central and/or peripheral distortion

**Fig. 12:**
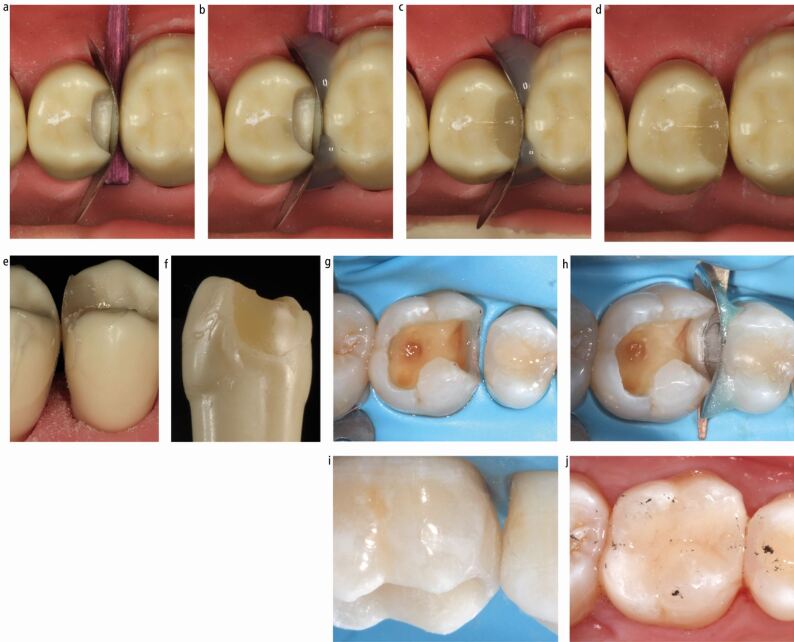
a, b, c, d, e, f) Rigid metal matrix separation and cervical stabilisation wooden wedge. Passive coronal stabilisation unbonded flowable composite. Good contact location and smooth convex surface to restoration at contact area. Minimal bucco-palatal excess accessible for finishing. g) Appropriate cavity design with all contact areas cleared. h) Wooden wedge providing apical stabilisation and separation. Rigid metal matrix and flowable resin providing passive coronal stabilisation. i, j) Good contact area, cervical and occlusal emergence achieved. Panels g, h, i and j courtesy of Christopher O'Connor

Active coronal stabilisation and separation with a separating ring can result in loss of a contact (Fig. 9) and/or peripheral and/or central distortion, which depends on the type of ring and placement technique, though the rings assessed in these studies are mostly outdated (Fig. 9).^16^^,^^21^^,^^27^ This potential exists with all designs of ring, in the author's experience (Fig. 10). The rings often create persistently tighter contacts than those occurring naturally, quite likely due to this distortion, suggesting the separation obtained is more than required.^13^^,^^28^ The peripheral and central distortion often results from a tendency of rings to tent the matrix, opening up gaps peripherally and forcing the contacting area against the adjacent tooth causing it to dimple in (Figures 9 and 10). Ultimately, different rings affect different matrices in different situations in different ways (Fig. 10), but then even the same ring, with the same matrix in the same situation, will result in different distortions even when placed by the same operator (Fig. 11). Therefore the technique, though it can be effective, has a level of unpredictability. These issues have led to the exploration of other methods to coronally stabilise sectional matrices in a more passive way, such as the use of unbonded flowable composite resin (Fig. 12), which reduces coronal stabilisation distortion.

### Extrusion distortion

Distortion can also occur when a matrix is insufficiently stabilised (coronally or cervically), during placement of uncured composite resin which is then able to extrude beyond the confines of a cavity. Pressure is therefore exerted on the moveable periphery of the matrix, potentially changing its shape, leading to peripheral and central distortion (Fig. 9).^21^ Anecdotally, flexible matrices are more susceptible to this distortion than rigid designs.

## Discussion

Concavities in the restoration at the contact area are often not visible clinically,^21^ so they will often not be identifiable after they have occurred. It is therefore critical to assess the matrix in terms of its adherence to the previously discussed objectives before placement of the restorative material.

Though distorted matrices can be burnished in an attempt to re-establish the shape at the contact area, this will always result in an uneven external topography to the resultant restoration if the matrix is made of metal. This may be less of an issue for clear matrices; however, the reason for the distortion (placement versus stabilisation) will impact on the ability for it to be easily resolved. Active stabilisation which results in central distortion cannot be simply resolved by burnishing because of the tenting mechanism of distortion.

Anecdotally, rigid metal matrices lend themselves to a degree of self-stabilisation coronally, which facilitates their use without a separating ring in many situations. When a ring is not used, separation with a firmly placed wooden wedge or mechanical separator is required. While plastic wedges and the Teflon-floss technique could potentially provide improved cervical adaptation in comparison with wooden wedges, their ability to separate teeth is currently uncertain. Although stiff matrices can have an element of coronal self-stabilisation, it is prudent to further stabilise the matrix with unbonded flowable composite (Fig. 12). This passive coronal stabilisation, allied with the minimised potential for extrusion distortion, likely increases the chances of obtaining an undistorted matrix. When an appropriately shaped matrix is chosen, this potentially results in an optimal cervical emergence and occlusal emergence out of the contact. There is also a smooth convex contact area in the resulting restoration with minimal ledging, accessible to finishing (Fig. 12). Flexible sectional matrices (≤50 microns thick) can also be stabilised coronally with this technique and can be used in larger cavities where walls are missing. It can however be technically more challenging to stabilise these matrices in the desired position. They often benefit from cervical stabilisation and sealing with PTFE tape following application of the wooden wedge, before coronal stabilisation with flowable composite. Further research to formally assess these issues would be beneficial.

## Conclusion

Sectional matrices are superior to circumferential matrices in terms of their ability to recreate lost interproximal walls in a seemingly more anatomical way. This has many potential patient-centred benefits, including the more predictable formation of contact areas which result in reduced reported food packing. It is apparent, however, that the achievement of a contact area could well be a Pyrrhic victory, if a clinically undetectable, inaccessible concavity in the restoration results in caries in the adjacent tooth.

Armed with an understanding of appropriate cavity design, where all contact areas are cleared, and the intricacies, advantages and limitations which exist for all of the various sectional matrices, methods of placement, stabilisation and separation are available, the practitioner can adopt a flexible approach. This will help to avoid many of the pitfalls associated with sectional matrix systems. Sectional matrices are susceptible to distortion at various stages in the restorative process. The practitioner should be aware of these issues to minimise their occurrence and to identify and address them before restoration placement, should they occur. This will engender confidence in obtaining predictable, anatomical contact areas resulting in improved patient-centred outcomes when restoring posterior interproximal cavities with direct composite.
